# Biomass partitioning and nutrient fluxes in *Silphium perfoliatum* and silage maize cropping systems

**DOI:** 10.1007/s10705-022-10242-0

**Published:** 2022-11-01

**Authors:** Thorsten Ruf, Christoph Emmerling

**Affiliations:** Department of Soil Science, Faculty of Regional and Environmental Sciences, University of Trier, Campus II, 54286 Trier, Germany

**Keywords:** Nutrient cycling, Soil organic matter, Root distribution, Cup plant, Perennials

## Abstract

**Abstract:**

Cup plant cultivation as feedstock for anaerobic digestion has become an emerging topic in European Agriculture. Although there is a gap in methane yields between cup plant and the benchmark crop silage maize, cup plant as a perennial crop provides several ecological advantages. Amongst others, studies have proven its potential for carbon sequestration. With the present study, we addressed the gap in knowledge about biomass partitioning above- and belowground as well as recycling of organic matter and nutrients for cup plant and compared the results to silage maize. Therefore, a 2 year field experiment was conducted under practical conditions on rather shallow soil conditions in a low mountain landscape in Western Germany. Relevant plant fractions like litterfall, yield biomass and stubbles were collected continuously and analyzed for their nutrient contents. Results show that the cup plant is characterized by more than 2000 kg ha^− 1^ a^− 1^ of pre-harvest losses with a high palatability. In sum, only 77% of the grown cup plant biomass can be harvested in contrast to 96% of silage maize. Thus, an intense, element-specific nutrient recycling takes place in cup plant whereas this is negligible in silage maize. Furthermore, clearly different, element-specific nutrient exports with yield were highlighted. In cup plant, exports were distinctly lower for nitrogen but several times higher for calcium compared to silage maize. Cup plant also showed 36% more roots with higher root masses particularly in the subsoil.

**Graphical abstract:**

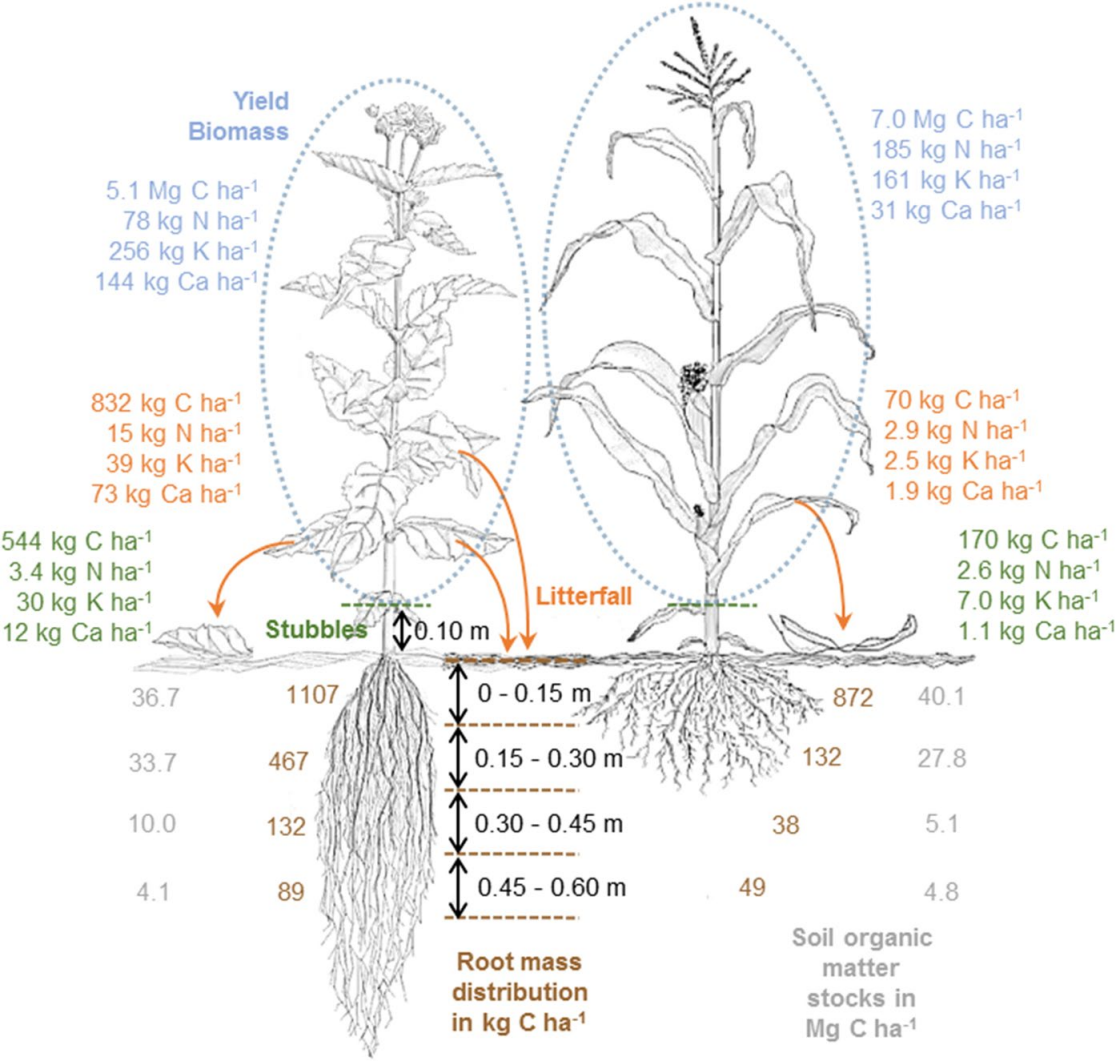

**Supplementary Information:**

The online version contains supplementary material available at 10.1007/s10705-022-10242-0.

## Introduction

The cup plant (*Silphium perfoliatum* L.) is one of the most promising perennial energy crops (PECs) under temperate conditions. In Central Europe, the cultivation area has increased by about 2000 ha per year during the past five years (A. Kipp, EPH, personal communication 14.09.2020). Until recently, the harvested biomass was predominantly used to replace silage maize as feedstock for anaerobic digestion. Cup plant may show biomass yields comparable to silage maize; however, concurring studies have shown that the biochemical methane potential is about 20 to 30% lower than that of silage maize (Haag et al. 2015; Mast et al. 2014; Ruf and Emmerling 2022; Schmidt et al. 2018). More recently, a sophisticated, sequential approach with a fiber separation to substitute wood in pulp industry followed by the anaerobic digestion of the residues is tested in pilot plants (Höller et al. 2021; Neis-Beeckmann 2021).

Several studies have highlighted the low-input character of PECs and their potential for carbon sequestration (Cumplido-Marin et al. 2020; Emmerling 2016; Ruf et al. 2018; von Cossel et al. 2019). Although there are significant reductions in management efforts for PECs, a recent study has proven that PECs harvested in a green state for anaerobic digestion purposes show significantly higher nutrient exports than PECs harvested in a brown state for thermal use (Ruf and Emmerling 2021). The potential of crops to accumulate soil organic carbon is a key issue in evaluating the sustainability of ‘energy from fields’ and significantly improving the greenhouse-gas balance of PECs compared to AECs (Adler et al. 2007; Brandão et al. 2011; Cadoux et al. 2014; Felten et al. 2013). Besides the withdrawal of carbon dioxide from the atmosphere, the soil organic matter is an essential component in order to maintain soil fertility (Blume et al. 2016). In general, two pathways for carbon entry into soils have to be distinguished. Firstly, there are distinct amounts of organic compounds that are released to the soil as root exudates (Carvalho et al. 2017). Secondly, the input of plant detritus at the end of the growing period from aboveground and belowground plant fractions, which is typically composed of root biomass, stubbles, and rather small amounts of shed leaves in agricultural systems, has to be taken into account (Balesdent and Balabane 1996; Johnson et al. 2014). According to Johnson et al. (2014), the belowground input of organic substances is of major relevance as it contributes to about two thirds to the soil organic carbon content. Several studies have claimed that PECs release significant amounts of pre-harvest losses that mainly consist of shed leaves (Anderson-Teixeira et al. 2013; Carvalho et al. 2017; Ruf et al. 2017). The emerging mulch layer possesses a couple of beneficial effects such as providing a source material for the formation of soil organic matter, covering the soil thus preventing evaporation losses and suppressing weeds, and presenting a nutrient reservoir e.g. for macro-saprophagous soil fauna (Ruf et al. 2017). Coincidently, perennial crops invest more efforts in the development of the rooting system, leading to a narrower shoot-to-root ratio, and allocate a higher share of assimilates belowground (Anderson-Teixeira et al. 2013; Carvalho et al. 2017).

In contrast to e.g. *Miscanthus* × *giganteus*, the biomass fractionation of cup plant has not been quantitatively assessed so far. In stands of cup plant, field observations revealed that leave wilting occurs starting from the stem basis presumably resulting from internal nutrient relocation likely mediated by low light intensities under the canopy. According to our observations, these wilted leaves are only weakly attached to the stalks and fall to ground during the vegetation period. On the contrary, studies of Schittenhelm et al. (2016) and Bury et al. (2020) report that the wilted leaves mainly remain at the stalks. However, after harvesting, a significant amount of plant residues could not be observed, except for stubbles. Thus, it has to be assumed that the litter was rapidly decomposed. The litterfall during the vegetation period may thus be an overlooked but meaningful plant fraction in carbon and nutrient recirculation in stands in cup plant.

Overall, there is a distinct lack of knowledge about the biomass fractionation as well as nutrient partitioning and fluxes in stands of cup plant. This part is essential for a better understanding of the ecology and physiology of stands of cup plant and may support an appropriate fertilization in practice. Moreover, it would help to gain insight in sources of organic carbon compounds leading to the outlined organic carbon storage in soil.

With this study, we aimed to compare cup plant and silage maize as benchmark feedstock for anaerobic digestion concerning biomass partitioning. We focused on aboveground and belowground plant fractions that contribute to carbon and nutrient fluxes and are thus also involved in nutrient recirculation and may stimulate carbon sequestration. Thus, we conducted an experiment in commercially managed stands of silage maize and cup plant. Pre-harvest losses (litter) were collected on a monthly basis and crop residues (stubbles) were accounted for after harvesting. After determining the yield biomass, also the belowground biomasses of both crops were determined in a sophisticated approach. All plant fractions were analyzed concerning their masses and macronutrient concentrations. Moreover, structural compounds of the plant fractions were determined in order to conclude for their degradability.

We hypothesized that there is a significant reduction of yield biomass resulting from pre-harvest losses caused by leave shedding in cup plant compared to silage maize. As the leaves fallen to the ground are not present any more at the time of harvest, we further assume that they show a chemical composition favorable for decomposition and are therefore contributing to nutrient recirculation. Moreover, we hypothesized that cup plant as perennial plant shows a higher root biomass compared to silage maize as annual crop.

## Materials and methods

### Experimental setup and site conditions

The study site was located in Western Germany about ten kilometers north of the city of St. Wendel at an altitude of 365 m a.s.l. The field experiments were conducted in neighboring stands of cup plant (established in 2017) and silage maize in the years 2019 and 2020. The fields were northerly exposed and showed an inclination of about 5°, which is typical for the situation of arable fields in low mountain ranges. The row-character of the stand of cup plant was still visible at the time of the field experiment.

As the stands were commercially managed by farmers, we established each five experimental plots within the stands of cup plant and silage maize about 30 m apart from each other and along the slope gradient. The plots were oriented perpendicular to the plant rows covering three of them (0.75 m row spacing) and were 2.00 m long thus having a size of 4.5 m².

According to FAO (2015), soils were classified as Hypereutric Cambisols (Aric Loamic Humic). In the topsoil (Ap horizon) down to 0.30 m, they showed a silt loam texture, pH values of 4.9 (in 0.01 molar CaCl_2_) and a bulk density of 1.45 g cm^− 3^. Subsoils were characterized by increasing clay contents, bulk densities and pH values resulting in loam texture, blocky angular soil structure and were weakly acidic. Starting from 0.65 m, slight mottling indicated adherent moisture. Parent material of soil development was a coarse sandy bedrock also containing pebbles from Cisuralian ages overlain with low amounts of Quaternary loess (Cohen et al. 2016; Geologisches Landesamt des Saarlandes 1989). Stone contents significantly increased starting at a depth of about 0.50 m. Thus, soils provide a maximum rooting depth of about 0.75 to 0.90 m, only.

Long term mean annual precipitation and temperature were 1031 mm and 9 °C (German Meteorological Service, 2022). Weather conditions in the experimental period were characterized by comparatively high precipitation in winters and above-average warm and dry summers relative to the long-term average (see supplementary material). N_min_ contents (0.0–0.90 m depth, extracted with 2 M KCl) prior to fertilization were 42 kg N ha^− 1^ and 38 kg N ha^− 1^ for cup plant and 89 kg N ha^− 1^ and 78 kg N ha^− 1^ for silage maize in the first and second year of the experiment, respectively. Both stands were fertilized in early spring of both years with 35 m^3^ ha^− 1^ of digestate amounting to about 150 kg nitrogen per hectare. In addition, the silage maize stand received 120 kg nitrogen in the form of calcium ammonium nitrate. Silage maize seeding was done after shallow tillage (0.12 m) using a cultivator. A single postemergence chemical weed control was applied in silage maize in each year. No plant protection measures were done in cup plant.

### Sampling and preparation of aboveground plant fractions

In the vegetation periods, the plots were visited on a monthly basis and litter already fallen to the ground as well as totally wilted leaves still adherent to the stalks were collected (Table 1). At the end of the growing season, the plants were harvested from the plots at a cutting height of 0.10 m, similar to the mechanical harvest that was conducted by the farmer some days later. Dry matter contents at harvest date were 29.1 (± 1.1) % and 30.7 (± 0.2) % for cup plant and 38.3 (± 1.2) % and 36.5 (± 2.9) % for silage maize in 2019 and 2020, respectively. The remaining stubbles were removed at ground level.

**Table 1:** Course of time of the experiment

Plant fraction	Cup plant	Silage maize
*2019*		
Litterfall 1st sampling	04.06.2019	02.09.2019
Litterfall 2nd sampling	05.07.2019	–
Litterfall 3rd sampling	09.08.2019	–
Litterfall 4th sampling	02.09.2020	–
Yield biomass	03.09.2019	16.09.2019
Harvest residues (stubbles)	04.09.2019	18.09.2019
Soil cores for root analysis	October 2019	October 2019
*2020*		
Litterfall 1st sampling	23.06.2020	19.08.2020
Litterfall 2nd sampling	22.07.2020	15.09.2020
Litterfall 3rd sampling	19.08.2020	–
Yield biomass	19.08.2020	15.09.2020
Harvest residues (stubbles)	21.08.2020	17.09.2020

All plant fractions were carried to the laboratory and dried at 45 °C in a compartment drier until constant weight. Dry masses were determined and the complete amount of sample (litter) or an representative subsample (yield biomass, stubbles) were milled using a cutting mill (Pulverisette 15, Fritsch GmbH, Idar Oberstein, Germany) to a particle size of 0.75 mm for further analyses.

### Sampling and preparation of belowground plant fractions

Sampling for roots was done after the first vegetation period in October 2019. Therefore, a manually driven soil core driller for root analysis (Eijkelkamp, Giesbeek, The Netherlands) with a diameter of 80 mm and a length of 150 mm, resulting in a volume of 0.75 L, was used. Due to the row structures of both crops, a standardized scheme accounting for the plant rows and interspaces was developed. Perpendicular to the plant rows, nine sampling points were realized per plot. The central sampling point was located in the center of the middle row of the plot. From this starting point, each four soil cores were sampled to the right and left side in a distance of each 0.25 m. Sampling was done down to a depth of 0.60 m with four consecutive samplings at the same place; below, a sampling was impossible due to high stone contents and bedrock material. In sum, the scheme resulted in 360 soil samples for root analysis.

Soil cores were stored in a refrigerator until root washing to impede rotting. Root washing followed the procedures of Böhm (1979). Therefore, soil cores were thawed overnight, crumbled by hand and then slurried in 10 L of water without any additives. Floating roots were collected from the water surface and the water decanted via a sieve tower with mesh sizes of 4  and 1 mm. Visible roots were collected from the sieves using forceps. Fresh water was added and the complete suspension poured again on the sieve tower and visible roots were collected. Oversize material was again suspended in water. These steps were conducted repetitively until no roots were visible. For final cleaning, the roots were washed in distilled water and then dried at 45 °C until constant weight which was finally determined.

For further macronutrient analyses, the amount of root sample was too small, at least for the lower depth. Therefore, all nine samples of a certain depth per plot were quantitatively pooled. The pooled samples were milled using an oscillating disc mill with metal inserts (Pulverisette 9, Fritsch GmbH, Idar-Oberstein, Germany).

### Analytical methods

Plant samples were analyzed for their nutrient contents in duplicates. Carbon and nitrogen determination was done simultaneously using an elemental analyzer (vario EL Cube, Elementar GmbH, Langenselbold, Germany). For the analysis of phosphorus (P), potassium (K), calcium (Ca), and magnesium (Mg), a total digestion was performed. Therefore, the milled plant samples were heat and pressure treated in a microwave system (Mars X CEM, GmbH, Kamp-Lintfort, Germany) after the addition of nitric acid (HNO_3_; 65%) and hydrogen peroxide (H_2_O_2_, 30%). Quantification of P was based on the colorimetric method of Murphey et al. (1962) and measured using a photometer (UV-1650 PC; Shimadzu Europe GmbH, Duisburg, Germany). Determinations of K, Ca, and Mg contents were done using a flame atomic absorption spectroscopy (AA240 FS, Varian GmbH, Darmstadt, Germany).

The composition of the aboveground plant fractions concerning acid detergent fiber (ADF), neutral detergent fiber (NDF) and acid detergent lignin (ADL) to estimate the structural components hemicellulose (NDF-ADF), cellulose (ADF-ADL), lignin (ADL) was determined according to the guidelines of VDLUFA (2012b, 2012c, 2012d) using a Fibertherm FT12-System (C. Gerhardt GmbH, Königswinter, Germany).

### Calculations and statistical analysis

The masses of the different aboveground plant fractions determined on a dry matter basis on the experimental plots (4.5 m²) were extrapolated to a reference size of one hectare. For the root biomasses, the volumes of the sampled soil cores per depth of an experimental plot (0.75 L and nine samplings per plot and depth equal to 6.75 L), were extrapolated to the volume of a soil layer with a size of one ha and a thickness of 0.15 m. For the calculation of nutrient contents of both, above- and belowground plant fractions, the concentration of a certain nutrient was multiplied with the dry matter mass of the respective plant fraction.

For statistical evaluation of the results, particularly high and low values were checked for plausibility. ‘Extreme’ values were not removed from the dataset as a very stringent and structured sampling was performed. Thus, it has to be assumed that the range of values observed represent the natural fluctuations resulting from slightly different soil conditions and heterogeneities occurring in agricultural management. Statistical evaluation targeted on comparing both crop species, silage maize and cup plant, to each other. Therefore, pairwise t-tests or Wilcoxon-tests were calculated depending on the preconditions ‘normal distribution’ and homoscedasticity’ which were initially checked using Shapiro-Wilk-Tests (Shapiro and Wilk 1965) and Levene-Tests (Levene 1960).

The statistical analysis and illustration was done using R programming language version 3.3.2 (R Core Team 2016).

## Results

### Aboveground biomass development, fractionation and nutrient partitioning

**Fig. 1:**
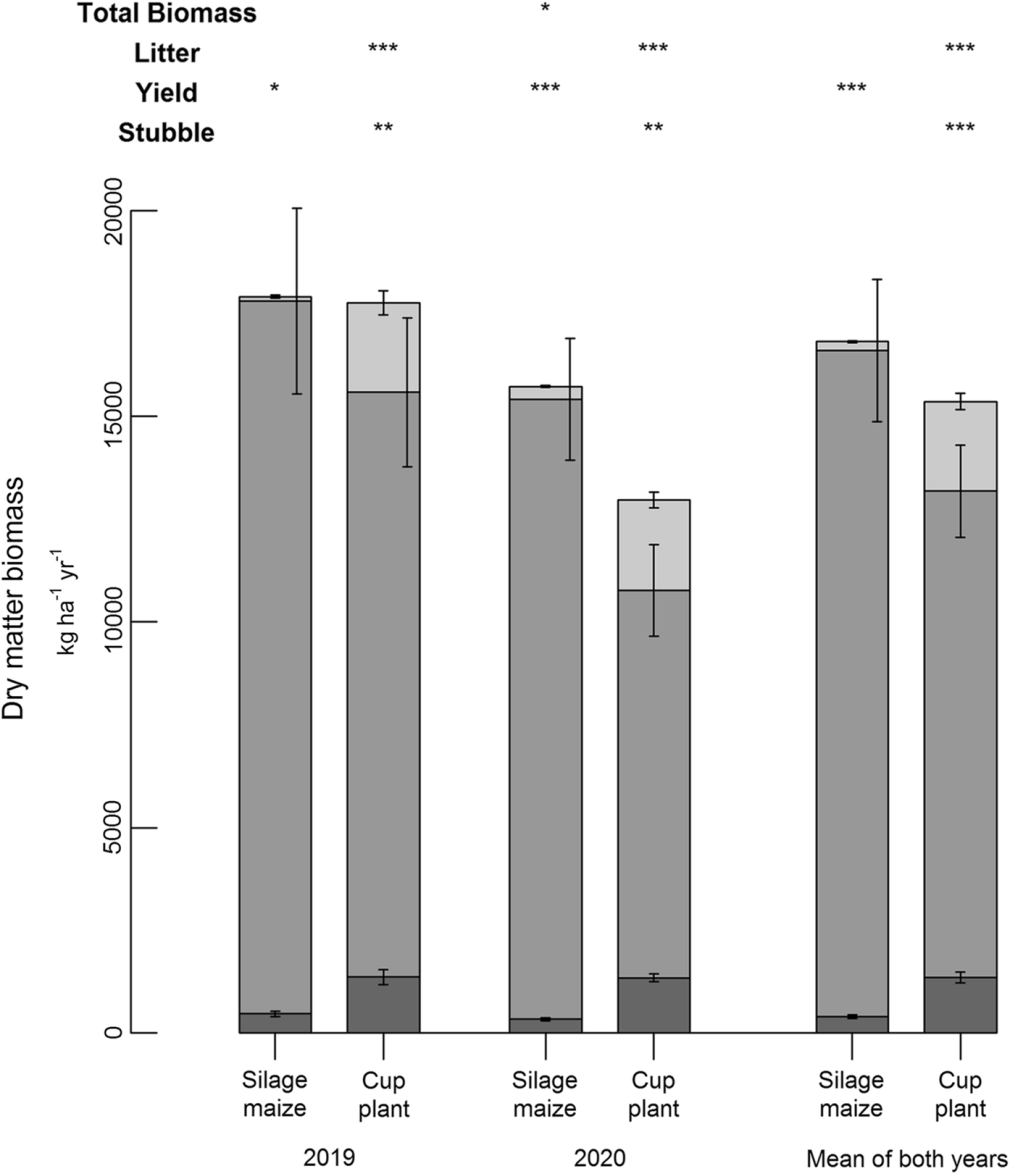
Aboveground biomasses determined in 2019, 2020 and as the mean of both experimental years subdivided into the plant fractions stubbles (undermost bar in darkgrey), yield biomass (middle bar in grey), and litter (uppermost bar in lightgrey) that comprises the sum of shed leaves of all samplings. ‘Total Biomass’ represents the sum of all three fractions. Significant differences between cup plant and silage maize are indicated as follows: ***: *p* < 0.001; **: *p* < 0.01; *: *p* < 0.05

Biomass development clearly correlated with the amount of precipitation in the vegetation period (see Supplementary Material) leading to significantly higher yields in 2019 compared to 2020. However, the yield decline of cup plant from 14,223 ± 1,812 kg d.m. ha^− 1^ (2019) to 9,427 ± 1,118 kg d.m. ha^− 1^ (2020) equal to 34% was much more pronounced than for silage maize that showed a reduction of only 13% (17,340 ± 2,256 kg d.m. ha^− 1^ in 2019 and 15,080 ± 1,479 kg d.m. ha^− 1^ in 2020) (Fig. 1). Apparently, the drier conditions of the year 2020 led to higher shares of leave shedding for both crops; however, the amounts of shed leaves were in general significantly higher (p < 0.001) in cup plant (2,183 ± 203 kg d.m. ha^− 1^, mean of both years) than in silage maize (213 ± 22 kg d.m. ha^− 1^, mean of both years). Similarly, the amounts of stubbles of cup plant (1,352 ± 136 kg d.m. ha^− 1^) were more than three times higher than that of silage maize (397 ± 43 kg d.m. ha^− 1^). The results revealed that the annual development of aboveground biomass of both crops did not statistically differ from each and amounted to 15,360 ± 1,327 kg d.m. ha^− 1^ and 16,820 ± 1,779 kg d.m. ha^− 1^ as mean of both years for cup plant and silage maize, respectively. Due to pronounced pre-harvest losses occurring particularly in drier years as well as the higher proportion of stubbles associated with lower yields, slightly more than 23 (± 1.0) % of the developed cup plant biomass but only 3.7 (± 0.2) % of silage maize were recycled.

For silage maize, the predominant fraction of recycling remained from the stubbles whereas in cup plant both fractions, stubbles and litterfall, contributed in the same magnitude to the recycling of biomass. However, element-specific differences were observed (Table 2). The total uptake (as sum of yield biomass, stubbles and pre-harvest losses) of nitrogen in cup plant (96.7 ± 15.9 kg ha^− 1^ a^− 1^) was only about 50% of the one of silage maize (190.9 ± 24.9 kg ha^− 1^ a^− 1^). In this context it has to be pointed out that nitrogen fertilization level of silage maize (270 kg N ha^− 1^ a^− 1^) was much higher than that of cup plant (150 kg N ha^− 1^ a^− 1^). Although this makes direct comparisons more difficult, it has to be considered that this study focussed on the assessment of different cropping systems under practice conditions and not on the abstract fact of similar fertilization levels. Nitrogen recycling in cup plant was mainly based on litterfall and that summed up to slightly more than 15 kg ha^− 1^ a^− 1^. In contrast, silage maize showed an equal distribution among stubbles and litter but on a significantly lower level with only 2 to 3 kg ha^− 1^ a^− 1^. 18.6 kg ha^− 1^ a^− 1^ equivalent to 19.6% and 5.6 kg ha^− 1^ a^− 1^ equivalent to 2.9% of the taken up nitrogen were recycled in cup plant and silage maize, respectively. Although the phosphorus uptakes of both crops were quite similar and amounted to about 22 to 26 kg ha^− 1^ a^− 1^, a different partitioning among the plant fractions became obvious. For both crops, the relative shares recycled were similar to that of nitrogen; however, stubbles and litterfall contributed to the same extent to it. Potassium uptake of cup plant (324 ± 27 kg ha^− 1^ a^− 1^) was twice that of silage maize (170 ± 17 kg ha^− 1^ a^− 1^) in mean of both years. However, almost 68  and 10 kg ha^− 1^ a^− 1^ equivalent to 21 and 5.6% of the total uptake were recycled in cup plant and silage maize, respectively. Cup plant was characterized by unique high uptake (229 kg ha^− 1^ a^− 1^), exports (146 kg ha^− 1^ a^− 1^) but also recycling (85 kg ha^− 1^ a^− 1^) of calcium. For all aspects, it was significantly higher (*p* < 0.001) than for silage maize with only 34.0, 30.7 and 3.0 kg ha^1^ a^− 1^ respectively. Litterfall was by far the most important source for recycling. The situation was similar for magnesium but on a lower absolute level.

**Table 2:** Element partitioning for pre-harvest losses presenting the sum of the litterfall during the vegetation period, the yield biomass and the harvest residues (stubbles) for both experimental years 2019 and 2020. The amounts of specific elements that remain at the field and are recycled are presented as mean values of both years in absolute (kg ha^-1^ a^-1^) and relative (%) figures based on the sum of aboveground plant fractions. Mean values ± standard deviation are presented. Significant differences between silage maize and cup plant are indicated as follows: ***: *p* < 0.001; **: *p* < 0.01; *: *p* < 0.05; °: *p* < 0.10

			Carbon		Nitrogen		Phosphorus		Potassium		Calcium		Magnesium	
			Silage maize	Cup plant	Silage maize	Cup plant	Silage maize	Cup plant	Silage maize	Cup plant	Silage maize	Cup plant	Silage maize	Cup plant
2019	Sum of litterfall	kg ha^− 1^ a^− 1^	38.5 (± 11.6)	829.0*** (± 80.5)	2.02 (± 0.78)	15.31*** (± 3.14)	0.18 (± 0.10)	2.37*** (± 0.37)	1.97 (± 0.47)	42.88*** (± 7.12)	0.75 (± 0.07)	66.91*** (± 10.50)	0.31 (± 0.07)	8.17*** (± 1.12)
	Stubbles		196.7 (± 32.0)	529.6*** (± 70.6)	3.41 (± 0.84)	3.34 (± 0.50)	0.14 (± 0.03)	2.47*** (± 0.48)	7.68 (± 0.51)	30.20** (± 4.59)	1.40 (± 0.39)	9.01*** (± 1.82)	0.96 (± 0.25)	1.36° (± 0.28)
	Yield biomass		6908.7 (± 407.7)	6197.2 (± 787.8)	208.04*** (± 33.07)	94.37 (± 28.80)	27.53 (± 4.55)	27.61 (± 4.10)	198.87 (± 27.76)	319.73** (± 72.13)	29.51 (± 7.77)	156.24*** (± 25.16)	23.93 (± 3.11)	31.66** (± 2.95)
2020	Sum of litterfall	kg ha^− 1^ a^− 1^	101.3 (± 13.1)	834.3*** (± 74.7)	3.84 (± 0.41)	15.12*** (± 1.41)	0.23 (± 0.03)	2.75** (± 0.39)	2.99 (± 0.29)	35.10*** (± 1.85)	2.86 (± 0.36)	79.79*** (± 7.05)	1.07 (± 0.11)	10.60*** (± 1.13)
	Stubbles		143.7 (± 13.9)	558.7*** (± 34.7)	1.84 (± 0.57)	3.49* (± 0.74)	0.02 (± 0.01)	3.54*** (± 0.79)	5.90 (± 2.23)	27.04*** (± 2.81)	0.89 (± 0.14)	15.27** (± 1.01)	0.57 (± 0.08)	2.27*** (± 0.39)
	Yield biomass		6755.2*** (± 669.7)	3945.0 (± 498.2)	162.72*** (± 19.03)	61.76 (± 7.46)	14.47° (± 0.59)	13.22 (± 1.28)	122.31 (± 20.59)	192.15** (± 28.08)	31.97 (± 4.22)	135.08*** (± 1.99)	25.08 (± 2.81)	27.99 (± 0.83)
Mean of both years	Sum in aboveground biomass	kg ha^− 1^ a^− 1^	7276.5° (± 788.3)	6446.8 (± 576.4)	190.94*** (± 24.89)	96.70 (± 15.88)	21.88 (± 3.31)	25.98° (± 2.09)	170.11 (± 16.84)	323.55*** (± 26.91)	33.73 (± 2.74)	228.88*** (± 18.20)	25.95 (± 1.73)	40.61*** (± 3.21)
	Amount of element recycled		240.1 (± 25.1)	1375.8*** (± 67.4)	5.56 (± 1.16)	18.63*** (± 1.22)	0.32 (± 0.12)	5.57*** (± 0.38)	9.52 (± 1.79)	67.61*** (± 3.07)	2.99 (± 0.36)	84.80*** (± 7.92)	1.45 (± 0.14)	11.20*** (± 1.24)
	Share of element recycled	% of sum in aboveground biomass	3.3 (± 0.2)	21.4** (± 1.1)	2.92 (± 0.56)	19.58*** (± 2.60)	1.46 (± 0.42)	21.46*** (± 0.91)	5.57 (± 0.68)	21.00*** (± 1.79)	8.91 (± 1.19)	37.11*** (± 2.85)	5.60 (± 0.40)	27.54*** (± 1.36)

### Structural compounds of aboveground plant fractions

The composition of the aboveground biomass concerning structural compounds showed distinct differences between cup plant and silage maize as well as among the plant fractions of a certain species (Table 3). In contrast, the variations between the years 2019 and 2020 were quite small. The shares of hemicellulose in all fractions of cup plant were quite low. In the litterfall, the values were steadily below the level of quantification (LOQ) and hemicellulose only accounted for about 5–10% of the yield biomass and stubbles, respectively. On the contrary, silage maize provided shares of 16–22% of hemicellulose with the higher values for yield biomass and stubbles. The shares of cellulose in litterfall were rather similar for cup plant and silage maize and typically ranged between 15 and 22%. Yield biomass of cup plant showed distinctly higher values of cellulose compared to silage maize. Inversely, the stubbles of silage maize showed higher shares of cellulose. However, the shares of cellulose of all plant fractions of both crops were lower in 2020 compared to 2019. Lignin contents for the litterfall fractions of both crops were in a range of 5–7%; similarly, no difference was observed for the stubbles with shares of 9–11% of lignin. In contrast to that, the shares of lignin in yield biomass amounted to more than 12% for the cup plant but were only about 5% for silage maize.

**Table 3:** Characterisation of the sampled plant fractions concerning their shares of structural compounds. Mean values ± standard deviation are presented. Values below the limit of quantification are indicated by ‘< LOQ’.

	Plant fraction	Species	Hemicellulose % of dry matter	Cellulose	Lignin
2019	Litterfall 1st sampling	Cup plant	< LOQ	18.4 (± 1.4)	7.2 (± 0.3)
		Silage maize	20.0 (± 1.5)	22.1 (± 3.6)	7.5 (± 3.8)
	Litterfall 2nd sampling	Cup plant	< LOQ	20.2 (± 2.4)	5.2 (± 1.0)
	Litterfall 3rd sampling	Cup plant	< LOQ	27.4 (± 1.0)	6.0 (± 0.4)
	Litterfall 4th sampling	Cup plant	2.2 (± 1.8)	15.2 (± 1.5)	11.6 (± 1.0)
	Yield biomass	Cup plant	5.7 (± 7.3)	29.6 (± 4.6)	12.8 (± 3.0)
		Silage maize	16.1 (± 1.6)	18.8 (± 2.2)	5.6 (± 0.8)
	Harvest residues (stubbles)	Cup plant	< LOQ	31.6 (± 2.5)	9.2 (± 0.4)
		Silage maize	16.6 (± 1.3)	41.3 (± 3.7)	10.1 (± 2.9)
2020	Litterfall 1st sampling	Cup plant	4.8 (± 2.8)	9.5 (± 3.6)	7.0 (± 1.7)
		Silage maize	16.8 (± 2.5)	9.8 (± 1.0)	3.8 (± 0.3)
	Litterfall 2nd sampling	Cup plant	1.6 (± 5.3)	15.3 (± 5.3)	5.7 (± 2.9)
		Silage maize	17.5 (± 3.4)	18.2 (± 5.4)	4.6 (± 0.7)
	Litterfall 3rd sampling	Cup plant	< LOQ	14.1 (± 9.3)	9.6 (± 1.6)
	Yield biomass	Cup plant	9.8 (± 1.1)	20.2 (± 1.8)	12.3 (± 1.2)
		Silage maize	21.8 (± 2.1)	9.8 (± 3.6)	4.3 (± 2.4)
	Harvest residues (stubbles)	Cup plant	10.5 (± 1.7)	20.6 (± 4.0)	11.7 (± 2.9)
		Silage maize	20.0 (± 1.6)	24.2 (± 2.2)	11.2 (± 1.4)

### Masses, depth gradients and nutrient contents of belowground biomasses

**Fig. 2:**
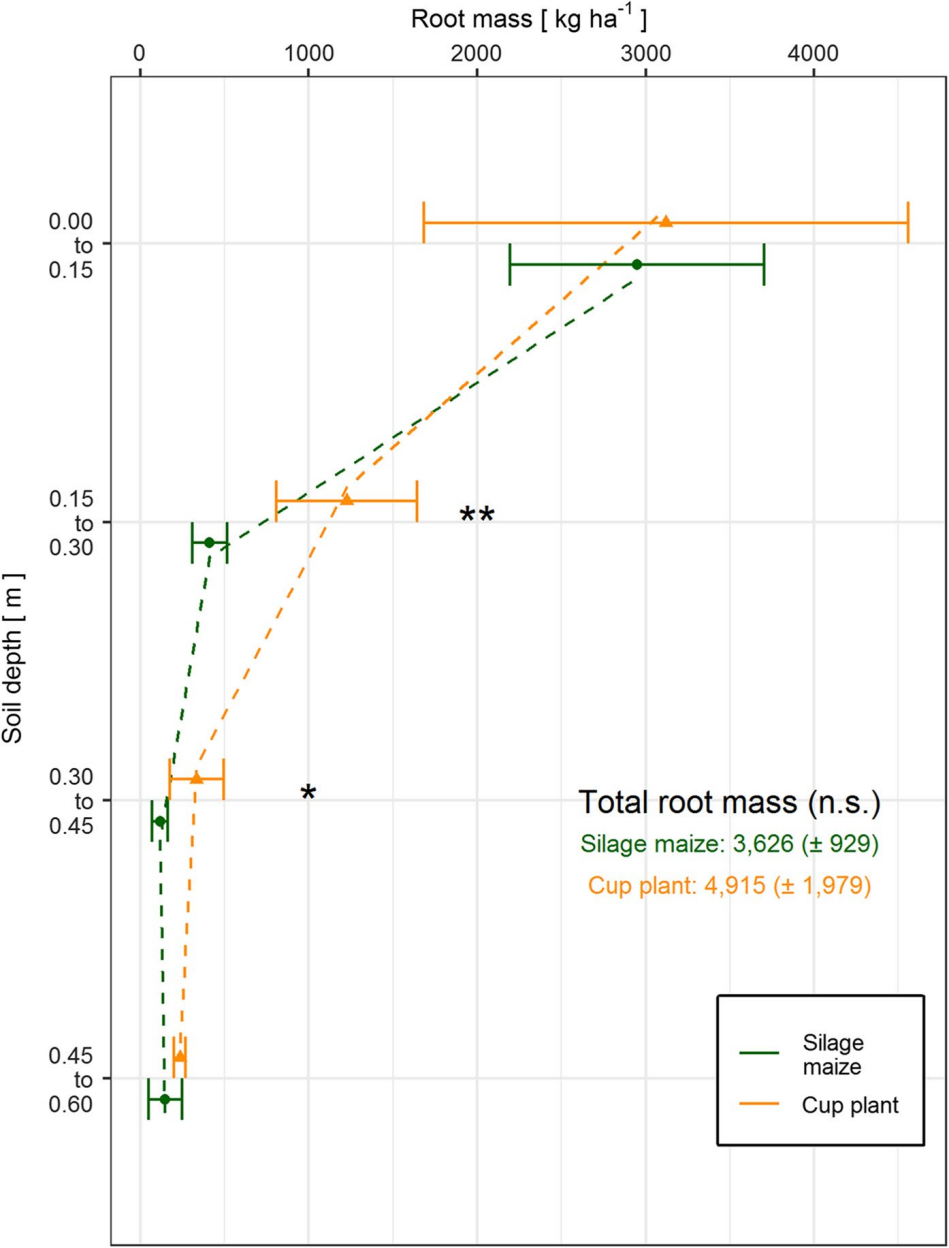
Root mass distribution of cup plant and silage maize in October 2019 as a function of soil depth for the depth intervals 0.00-0.15 m, 0.15–0.30 m, 0.30–0.45 m, and 0.45–0.60 m. Mean values ± standard deviation are illustrated. Statistical differences between cup plant and silage maize at a certain depth interval are indicated as follows: **: *p* < 0.01; *: *p* < 0.05

The total belowground biomasses showed no significant difference between cup plant (4,915 ± 1,979 kg ha^− 1^) and silage maize (3,626 ± 929 kg ha^− 1^). However, despite the stringent and standardized sampling procedure for the roots, the differences among the replicates were rather large (Fig. 2). By comparing the five replicates for cup plant, the total root mass ranged from 2,409 kg ha^− 1^ to 7,291 kg ha^− 1^. The range of values observed for silage maize was lower (2,391 kg ha^− 1^ and 4,843 kg ha^− 1^). Particularly in the topmost horizon, the differences among the replicates were large for both crops. Nonetheless, the root masses of cup plant were higher in all depths being significant in the depth intervals of 0.15–0.30 m and 0.30–0.45 m. A total of 93% of the root biomass of silage maize was found in the Ap horizon (0.00-0.30 m), whereas the share of cup plant roots was lower (88%) indicating a more intense rooting system in the subsoil. The decline in root biomass from the uppermost soil layer (0.00-0.15 m) to the underlying one (0.15–0.30 m) was much more pronounced in silage maize than in cup plant. The second layer contained slightly more than one-third of the root mass of the topmost one in cup plant but only one-sixth in silage maize.

Amounts of nutrients stored in the root biomass generally followed the root mass distribution as no significant differences in the nutrient concentrations among the different soil depths were determined. Thus, about 90% of the nutrients were stored in the roots of the Ap horizon (Table 4). The total amounts of single macronutrients down to a depth of 0.60 m were always higher for cup plant than for silage maize. About 84 ± 41  and 57 ± 13 kg ha^− 1^ of nitrogen were fixed by the roots of cup plant and silage maize, respectively. Similar to aboveground biomass, the amount of potassium (28.6 ± 10.9 kg ha^− 1^) and particularly calcium (35.1 ± 18.3 kg ha^− 1^) in the roots of cup plant were distinctly higher compared to silage maize (22.7 ± 6.1 kg K ha^− 1^ and 14.3 ± 3.4 kg Ca ha^− 1^, respectively). In contrast, phosphorus and magnesium were on an equal level for silage maize and cup plant being in a mid-single digit range.

**Table 4:** Mean values ± standard deviation of nutrient contents of root biomass in different depths. Significant differences between silage maize and cup plant are indicated as follows: ***: *p* < 0.001; **: *p* < 0.01; *: *p* < 0.05; °: *p* < 0.10

Depth	Species	Carbon	Nitrogen	Phosphorus	Potassium	Calcium	Magnesium
m		kg ha^−1^					
0.00–0.15	Cup plant	1107.19 (± 600.08)	58.90 (± 34.75)	3.40 (± 1.63)	18.60 (± 8.43)	22.28 (± 12.77)	6.19 (± 2.91)
	Silage maize	871.89 (± 202.48)	48.51 (± 11.40)	3.04 (± 0.75)	18.33 (± 4.71)	12.11 (± 3.17)	6.56 (± 1.70)
0.15–0.30	Cup plant	466.98* (± 194.33)	18.38** (± 4.96)	1.17** (± 0.35)	6.66** (± 2.06)	8.88* (± 4.51)	2.30** (± 0.64)
	Silage maize	132.24 (± 35.91)	5.39 (± 1.59)	0.33 (± 0.08)	2.31 (± 0.93)	1.46 (± 0.38)	0.81 (± 0.30)
0.30–0.45	Cup plant	131.88* (± 74.15)	4.09* (± 1.92)	0.29 ° (± 0.18)	1.92* (± 0.77)	2.43* (± 1.37)	0.59* (± 0.22)
	Silage maize	37.63 (± 15.80)	1.31 (± 0.58)	0.07 (± 0.02)	0.91 (± 0.27)	0.33 (± 0.11)	0.26 (± 0.08)
0.45–0.60	Cup plant	88.63 ° (± 19.82)	2.59° (± 0.32)	0.18** (± 0.02)	1.45 (± 0.27)	1.45*** (± 0.27)	0.42 (± 0.08)
	Silage maize	48.65 (± 34.13)	1.54 (± 1.06)	0.08 (± 0.05)	1.11 (± 0.74)	0.34 (± 0.23)	0.33 (± 0.21)
Sum 0.00–0.60	Cup plant	1794.68 (± 858.58)	83.96 (± 40.60)	5.05 (± 2.14)	28.63 (± 10.94)	35.05 ° (± 18.28)	9.50 (± 3.64)
	Silage maize	1090.40 (± 255.08)	56.74 (± 12.71)	3.52 (± 0.80)	22.67 (± 6.07)	14.25 (± 3.39)	7.96 (± 2.10)

## Discussion

### Leave shedding in cup plant leads to biomass losses and nutrient cycling

Whereas the biomass development of both crops was similar in 2019, cup plant obviously suffered from limited soil water availability during early summer in the distinctly drier year 2020. This aspect can be explained by the results of Schoo et al. (2017a) who stated a 34% lower water use efficiency of the C3-crop cup plant compared to silage maize being a C4-plant. In both years, cup plant has shown leaf shedding prior to harvest about ten times higher than silage maize. This likely results from increasing shading of the lower plant sections during canopy closure and height development due to the very high maximum leaf area index (LAI_max_) of about ten as estimated by Schittenhelm et al. (2016). They state that already to the middle of June, the leaves at the lower stem sections were wilted and not photosynthetically active any more. In contrast to the present study, they observed that the wilted leaves remained at the stalks until harvest. Moreover, they also observed that under drier conditions, in their treatment without additional irrigation, the reduction in leaf area was even more pronounced. Bury et al. (2020) outline LAI-values from 4.79 m^2^ m^− 2^ to 5.15 m^2^ m^−^d>^2^. They characterized the processes of stalk density reduction and wilting of leaves as “self-regulation of the canopy”. Similar to Schittenhelm et al. (2016), Bury et al. (2020) describe that at the end of the vegetation period (end of September), ‘some of the lower leaves had fallen off completely, and some of the leaves were dried, brownblack colored, especially in the middle part of stem’. At harvesting dates in winter for combustion purposes, Bury et al. (2020) observed that ‘Silphium biomass at the time of harvest […] consists almost entirely of stems’. It has to be highlighted that the observations of Schittenhelm et al. (2016) and Bury et al. (2020) concerning leave senescence, wilting and shedding did not match those of the present study. Reasons for these differences are not obvious; they may be found in different accession or soil characteristics. Moreover, the used approach for leave collection by ground sampling of already fallen leaves as well as removing completely wilted, weakly attached leaves from the stems in this study certainly led to slightly method-related results. Thus, the values presented in this study concerning leave shedding and associated biomass reduction as well as nutrient recycling should be regarded as an upper limit.

The number of cup plant stubbles determined in this study was distinctly higher compared to silage maize by the fact that numerous shoots emerged from each rootstock of cup plant. In sum, 2183 kg d.m. ha^− 1^ of litter and 1352 kg d.m. ha^− 1^ of stubbles, equivalent to 23.1% of the aboveground biomass, have to be classified as pre-harvest losses and yield remainings that significantly reduced the amount of yield biomass. Contrarily, the yield reduction for silage maize was only 3.6% (213 kg d.m. ha^− 1^ of litter and 397 kg d.m. ha^− 1^ of stubbles). Thus, the harvest index (economic yield) of cup plant was significantly lower than for silage maize (76.9% vs. 96.4% as mean of both years). Due to the large amounts of pre-harvest losses in cup plant, that may also occur in other perennial crops such as Miscanthus with masses between 1500 and 5000 kg d.m. ha^− 1^ (Amougou et al. 2011; Mangold et al. 2019; Ruf et al. 2017), this fraction urgently needs to be addressed in the calculation of the harvest index for perennial energy crops, although it is commonly only defined as developed aerial biomass minus stubbles (Donald and Hamblin 1976).

From an economic point of view, a significant yield reduction in stands of cup plant has to be seen as a severe problem with respect to competitive profits; particularly as the biochemical methane potential is also about 20 to 30% lower than that of silage maize (Haag et al. 2015; Mast et al. 2014; Schmidt et al. 2018). Overall, cup plant provides methane yield per hectare 30–40% lower than silage maize under these site conditions (Ruf and Emmerling 2022). This economically unprofitable aspect of cup plant cultivation coincidently reveals one of its main ecological and environmental advantages. Resulting from the large amounts of organic residues, a distinct potential for the stimulation of soil life, carbon sequestration and nutrient recycling arises. However, as the composition of structural compounds (Table 3) is quite different among the plant fractions, a differentiated view of the different recycled plant factions is necessary. The stubbles are highly lignified and poor in nutrients (Tables 2 and 3). Moreover, their upright position remains, to our observation, at least until the summer of the following year. Although weathering is visible and certain soluble components will likely be leached, it has to be assumed that the stubbles do not significantly contribute to the soils’ food web and carbon sequestration in cup plant cultivation. Although the palatability and digestibility of silage maize stubbles is as unfavourable as that of cup plant, soil tillage measures lead to a better soil contact and much faster decomposition. This aspect is of relevance to reduce the potential of plant pathogens or pest organisms. Even through there are no distinct yield reducing issues known so far for cup plant (Gansberger et al. 2015), this may become an emerging issue in future with expanding of the cultivation area.

In contrast, the shed leaves of both crops are easily decomposable as they mainly consist of cellulose, are rich in nutrients and thus show a narrow C to N ratio. However, input of organic substances by litterfall is only of relevance in cup plant as the amount of shed leaves in silage maize is negligible. Although leave shedding takes place in summer with a lack of precipitation, the decomposition of the litter proceeds very rapid. The large amounts of organic residues at the soil surface in perennial energy crops in combination with the absence of tillage present valuable habitat conditions for soil flora and particularly macro-fauna like earthworms (Emmerling et al. 2021; Schorpp and Schrader 2016). Moreover, the damp and warm microclimate after canopy closure, resulting from (i) high transpiration rates as long as soil water availability given (Schoo et al. 2017a) and (ii) capillary water rise combined with reduced evaporation due to the shaded soil surface, accelerates the decay of organic residues at the soil surface. With respect to the last-mentioned point and by general ignorance of the significance of the mentioned processes it appears questionable if the litter is of relevance for carbon sequestration as the predominant part of decomposition obviously takes place at the soil surface. Nonetheless, in all cases, a fast in-season nutrient remobilization appears likely. This may be one factor why cup plant may be able to show respectable biomass yields on soils with a low nutrient supply.

### More intense rooting of deeper soil layers by cup plant

The study revealed about 36% higher (*p* = 0.24) total root masses for cup plant. Despite the strict sampling design and the huge number of replicates, the standard deviations were still high. This convincingly shows the important role of large sampling sizes for analysis of belowground biomass in row crops, especially in low mountain landscapes with shallow, skeleton-rich soils that do not allow for a homogeneous rooting in subsoil. Particularly in these layers, the root masses of cup plant were significantly higher for cup plant compared to silage maize (Fig. 2). The explanatory power of root masses concerning absorption capacity of roots is limited compared to specific root length density (SRL) (Fitter 1985, 1991). SRL could not be quantified in this study. However, as observed during root washing, the root mass of the lowest depth analysed (0.45–0.60 m) was predominantly made up of fine roots showing a good soil volume exploitation. This indicates for a good ability to adsorb soil water and nutrients in subsoil. Nonetheless, the limited rooting depth of the study sites, which is below one meter, distinctly limits the available soil water resources. By this fact, the results of this study cannot be directly compared to those of Schoo et al. (2017b), as they described the root system under distinctly different, mostly deep soil conditions with low shares of soil skeleton. In contrast, root growth was limited in depth by the massive bedrock present at our study sites starting at depths of about 0.75 m. However, similar to our study, also Schoo et al. (2017b) found an exponential decrease in root mass with depth for both crops but also a more intense rooting of deeper soil layers by the cup plant.

Our results are in line with studies (Ende et al. 2021; Ruf and Emmerling 2018; Schoo et al. 2017a) that have refuted the initial assumption that the cup plant suits very well for cultivation under drier soil conditions (Bauböck et al. 2014; Pan et al. 2011; Sokolov and Gritsak 1972). Although Schoo et al. (2017b) have shown that cup plant has not a higher water capture capacity than maize, the enduring rooting system in the subsoil may be favorable. Likely, the vulnerable to spring drought is much lower than for silage maize leading to a faster biomass development early in the vegetation period which finally may lead to the pleasing biomass yields observed. Compared to silage maize as C4-crop with a water use efficiency of 50 kg ha^− 1^ mm^− 1^ being 50% higher than that of cup plant (33 kg ha^− 1^ mm^− 1^) (Schoo et al. 2017a), the aboveground biomass development of cup plant (15,360 kg d.m. ha^− 1^, mean of both years) was only 8.7% lower than that for silage maize (16,820 kg d.m. ha^− 1^, mean of both years), regardless the distinct lack of precipitation in the summers of both experimental years. The feature of a permanent, in spring already deeply developed rooting system is certainly a major advantage of perennial over annual crops with respect to climate change with a predicted increasing frequency of severe spring dryness (Jacob et al. 2014). The distinctly narrower shoot-to-root ratio of cup plant (3.1) compared to silage maize (4.6) indicates that cup plant allocates more assimilates to belowground biomass; a behavior typical for perennial crops (Carvalho et al. 2017), as roots are essential organs for the survival of (Hemi)Crypthophytes (Ellenberg 1967). High root masses may come along with the ability to foster soil microbial activity due to the excretion of low-molecular substances as easily available carbon source. This may also be the reason for the observed increase in soil organic carbon contents under cup plant cultivation (Emmerling 2016, Ruf and Emmerling 2020). In contrast, the contribution of aboveground biomass fractions (stubbles and litterfall) to soil organic matter formation appears questionable despite the large amounts and good palatability. To our observation, the decomposition predominantly takes place at the soil surface as biotic incorporation (e.g. by the activity of earthworms) proceeds rather slow. These assumptions are supported by a density-fractionation-based analysis of the soil organic matter composition and quality carried out by Ruf and Emmerling (2020). They found that under cup plant cultivation, particularly the fraction of occluded particulate organic matter (oPOM) with a density between 1.6 and 2.0 g cm^− 3^ was increased while the changes in free particulate organic matter (fPOM) was of minor relevance. Similar results were described by Kantola et al. (2017) who found no significant change in total soil organic carbon content under perennial crops (*Miscanthus *×*giganteus* , Switchgrass), but an enrichment of the POM-pools. Whereas the fPOM fraction results from mechanically shred, incorporated plant detritus of low quality (like the stubbles), the oPOM fraction results from more easily degradable organic matter, like roots and exudates that are rapidly incorporated and protected in a physical way (cf. Rasse et al. 2005). These assumptions are further supported by the analysis of soil samples from the experimental plots (data not shown) taken in two depth (0.00–0.125 m and 0.125–0.25 m) at the times of litterfall collection. The mean values (several samplings per year) of the microbial biomass did not differ between both crops neither in the upper nor in the lower depth; however, the microbial activity determined as basal respiration was significantly higher under cup plant in both depth with the most distinct difference in the depth of 0.125–0.25 m (0.25 to 0.38 µg CO_2_-C g^− 1^ h^− 1^). However, it has to be taken into account that soil data from a non-tillage system (cup plant) are compared to a reduced tillage system (silage maize) are compared here.

Carbon accrual by (i) root exudation and (ii) root mass turnover with deep rooting crops may present the benefit of a higher stabilization of the soil organic carbon resulting from physical protection due to the larger distance to the soil surface with unfavorable conditions for microbial degradation processes. After a possible shift in land-use back to annual crops, the turnover of soil organic matter in subsoil is hypothesized to be much slower than in the tilled topsoil leading to a long(er) lasting carbon sequestration.

### Cup plant requires adjusted soil management

The results of the present study convincingly show that adapted management strategies for cup plant need to be established. With respect to the targeted durability of 15 to 20 years for stands of cup plant, the specific needs concerning nutrients have to be addressed. As shown by comparing cup plant and silage maize on neighbouring stands under similar soil and weather conditions, the structure of nutrient exports and thus the demand for compensation significantly differ between both crops (Table 2). Likely, due to the absence of a large storage organ like the cob in silage maize and the demonstrated nitrogen recirculation particularly by leave shedding, the nitrogen exports of cup plant are less than 50% of that of silage maize. Although nitrogen demands for the accrual of soil organic matter under cup plant have to be taken into consideration, reduced nitrogen fertilization should be encouraged. Nitrogen fertilization should be done in early spring as the cup plant shows a very fast flushing with a high nutrient demand. In contrast, a fertilization in autumn, at least in low mountain landscapes, as to date commonly conducted, has to be avoided as no secondary flushing after the harvest was observed, presumably due to dry soil conditions followed by cool temperatures. Together with the deep and intense rooting system, the forgoing of nitrogen fertilization in autumn will lead to low contents of soil mineral nitrogen and diminish the leaching potential.

For potassium and calcium, the situation is contrary. The exports of potassium in mean of both years were 160 kg ha^− 1^ a^− 1^ and 256 kg ha^− 1^ a^− 1^ for silage maize and cup plant, respectively. An even more pronounced difference was observed for calcium with the exports for cup plant (146 kg ha^− 1^ a^− 1^) being five times higher than for silage maize (31 kg ha^− 1^ a^− 1^). Similar results, however expressed as nutrient demand per ton dry matter yield were presented by Ustak et al. (2018). Also compared to other agricultural crops the calcium contents and exports of cup plant were unique in height (Schilling 2000). It is not clear if the high uptake is demand-driven or simply an accumulation of cations taken up with mass flow resulting from high transpiration rates (Schoo et al. 2017a).

High calcium exports imply a significantly increased demand for liming. Typically, an amount of 500 kg Ca^2+^ ha^− 1^ is annually required to compensate for leaching and export with yield of agricultural crops typically leading to a liming of 1500 kg Ca^2+^ ha^− 1^ after a rotation period of three years. During cup plant cultivation, this amount certainly needs to be elevated to about 2000 kg Ca^2+^ ha^− 1^. Attention has to be paid that the soil pH will not drop in the course of cup plant cultivation. This fact will further put a strain to the economic balance sheet of cup plant cultivation, additionally to the reduced revenues due to the distinctly lower methane yield per hectare (Ruf and Emmerling 2022).

Summarizing, a compensation of the nutrient exports with digestate, as typically done, will not meet the requirements of cup plant. For fertilization planning, the first objective is to meet the nitrogen demand of the crop. According to KTBL (2012), the ratio of N-P-K-Ca-Mg of digestates from cattle manure fermented with biomass feedstocks is about 1-0.24-0.96-0.34-0.12. The nutrient exports of silage maize show a ratio of 1-0.12-0.87-0.17-0.13; thus, the compensation of the nutrient exports succeeds very well. In contrast, for cup plant the nutrient export ratio (1-0.26-3.28-1.85-0.38) and the contents in digestate show a large discrepancy. The cup plants’ demands for K, Ca and Mg can by far not be compensated by simple recycling of digestates from biogas plants largely fed with silage maize. Coincidently, the significantly lower nitrogen exports with cup plant compared to silage maize have to be considered in order to perform environmental-friendly fertilization. Thus, adapted fertilization strategies need to be developed for cup plant cultivation instead of simply transferring the ones used for silage maize.

## Conclusion

The study has revealed and quantified the biomass partitioning and fluxes of organic matter and macronutrients in stands of cup plant. In contrast to silage maize, the cup plant shows an intense cycling of organic matter from aboveground plant fractions that can easily be decomposed resulting from their low degree of lignification. As observed, the high transpiration rates of cup plant, as long as sufficient soil water availability is given, lead to a damp microclimate under the cup plants canopy accelerating the litter decomposition under warm temperatures in summer. Although the fast decomposition of the leaf litter certainly releases nutrients that are readily available for uptake, there are doubts regarding the effects on carbon accrual by these plant fractions as the decomposition occurs predominantly at the soil surface. More likely, the intense rooting system contributes to carbon sequestration and furthermore, in combination with the large amounts of recalcitrant stubbles, these are keys to prevent soil erosion. Thus, the cultivation of cup plant as feedstock for anaerobic digestion shows beneficial effects for soil protection and carbon sequestration. Still, cup plant as an agricultural crop is a more recent evolution involving many unknown variables regarding suitable stand management. In agricultural practice for example, the specific nutrient demand of cup plant needs to be addressed in order to (i) maintain soil fertility (ii) prevent losses of plant nutrient to the environment and (iii) to reach the expected life span of the crop which is estimated to be 15 to 20 years.

## Electronic supplementary material

Below is the link to the electronic supplementary material.

Supplementary Material 1

## Data Availability

The datasets generated during and analysed during the current study are not publicly available but are available from the corresponding author on reasonable request.
