# Supplementary material for: Biomass partitioning and nutrient fluxes in Silphium perfoliatum and silage maize cropping systems
Source: Nutr Cycl Agroecosyst. 2022 Nov 1;124(3):389–405. doi: 10.1007/s10705-022-10242-0 (PMC9628351; doi:10.1007/s10705-022-10242-0)

**Supplementary Material**:

Supplementary Figure 1: Representation of the climatic conditions of the experimental period. Data retrieved from the German Meteorological Service (DWD, 2021) for the station ‘Tholey’ (station id: 5029) located approx. six kilometres SW of the study sites.


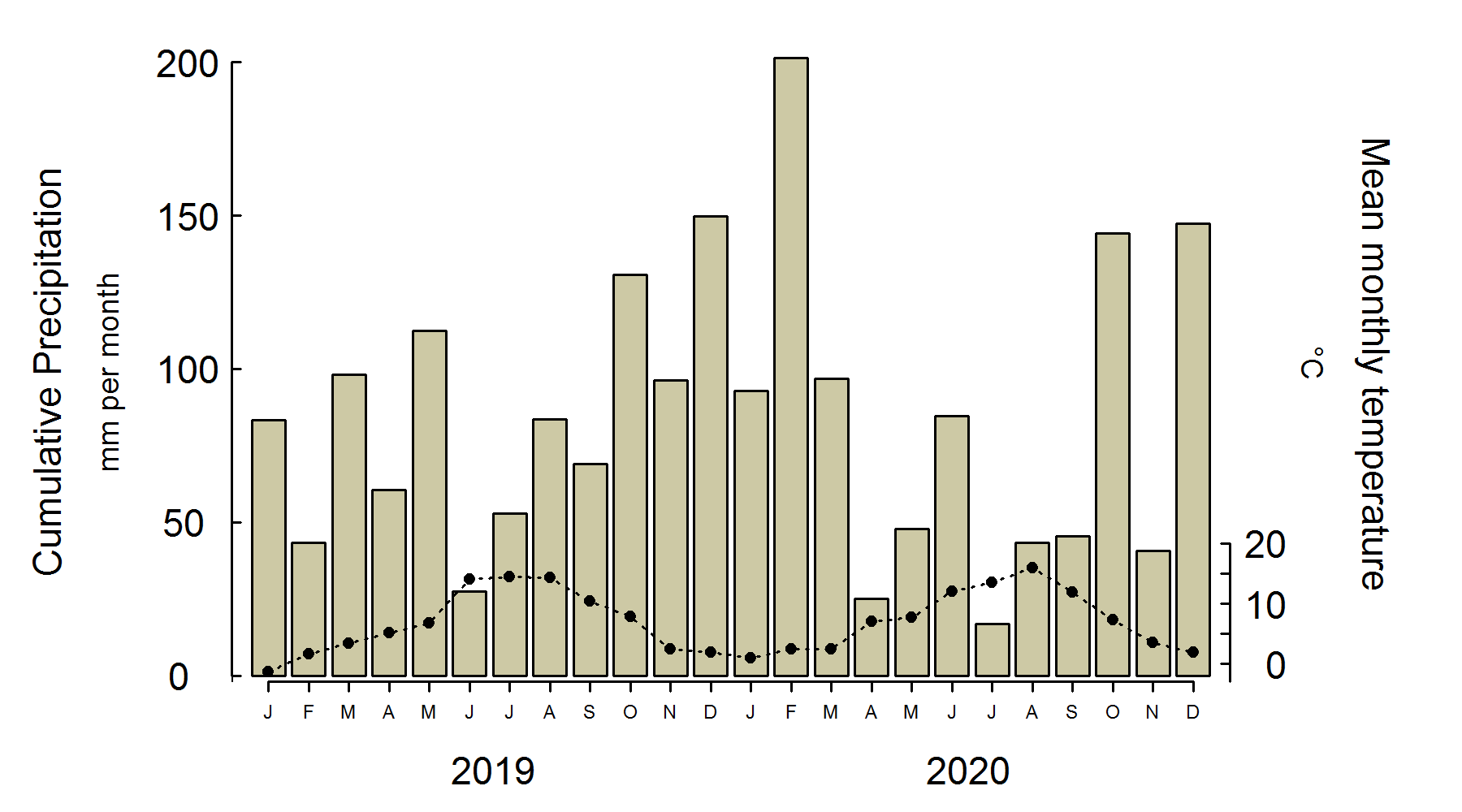

Supplement: Supplementary file 1 — Supplementary Material 1 [file 10705_2022_10242_MOESM1_ESM.doc]
